# Syphilis

**Published:** 1891-10

**Authors:** 


### Syphilis.

Primary Symptoms. —About three weeks after contagion (sometimes longer), an erosive indurated papule, usually single, painless and non-recurrent, with or without ulceration or discharge, generally attacks the glands or prepuce in men, and labia or nymphæ in women.

About two weeks later (or less), painless induration and enlargment of inguinal glands without suppuration. Cervical, axillary and other glands may enlarge later on.

The above symptoms (true chancre and syphilitic bubo) are always followed by constitutional syphilis.

Secondary Symptoms.—About six weeks (more or less), after contagion, a roseolous rash on chest or abdomen, with erythema or ulceration of fauces and larynx, succeeded by papular (lichen), vesicular (eczema), or pustular (ecthyma, acne, impetigo) eruptions of body, fauces, tongue and tonsils, and mucous patches and condylomata of orifices.

About three months later, squamous (psoriasis, lepra) or bullous (rupia, pemphigus), eruptions of the skin and fauces. Rheumatoid and periosteal pains. Iritis, choroido-retinitis. Temporary deafness. Partial alopecia. Epididymitis. Onychia of fingers and toes.

Tertiary Symptoms.—About three or four years after contagion (sometimes much sooner), serpiginous ulcerations of skin and mucous membranes. Pigmentary syphilides. Rupia. Destructive onychia. Nodes, caries, necrosis, and exostosis of tibia, ulna, sternum, and cranial or nasal bones. Hard, painless orchitis. Scirrhus. Permanent deafness. Gummy tumors of skin, mucous membranes, liver, lungs, kidneys, tongue, palate, uterus, joints, muscles, tendons, brains and spinal cord. Epilepsy. Hemiplegia. Paraplegia. Paralysis limited to certain muscles. Dactylitis. Stricture of pharynx and esophagus.

Should a dentist use intoxicants; Better not. It is an injustice to his patients, and worse for bimself.

